# Modulation of Self-Esteem in Self- and Other-Evaluations Primed by Subliminal and Supraliminal Faces

**DOI:** 10.1371/journal.pone.0047103

**Published:** 2012-10-16

**Authors:** Ran Tao, Shen Zhang, Qi Li, Haiyan Geng

**Affiliations:** 1 Department of Psychology, Peking University, Beijing, China; 2 Department of Psychology, University of Wisconsin-Whitewater, Whitewater, Wisconsin, United States of America; University of Minnesota, United States of America

## Abstract

**Background:**

Past research examining implicit self-evaluation often manipulated self-processing as task-irrelevant but presented self-related stimuli supraliminally. Even when tested with more indirect methods, such as the masked priming paradigm, participants' responses may still be subject to conscious interference. Our study primed participants with either their own or someone else's face, and adopted a new paradigm to actualize strict face-suppression to examine participants' subliminal self-evaluation. In addition, we investigated how self-esteem modulates one's implicit self-evaluation and validated the role of awareness in creating the discrepancy on past findings between measures of implicit self-evaluation and explicit self-esteem.

**Methodology/Principal Findings:**

Participants' own face or others' faces were subliminally presented with a Continuous Flash Suppression (CFS) paradigm in Experiment 1, but supraliminally presented in Experiment 2, followed by a valence judgment task of personality adjectives. Participants also completed the Rosenberg Self-Esteem Scale in each experiment. Results from Experiment 1 showed a typical bias of self-positivity among participants with higher self-esteem, but only a marginal self-positivity bias and a significant other-positivity bias among those with lower self-esteem. However, self-esteem had no modulating effect in Experiment 2: All participants showed the self-positivity bias.

**Conclusions/Significance:**

Our results provide direct evidence that self-evaluation manifests in different ways as a function of awareness between individuals with different self-views: People high and low in self-esteem may demonstrate different automatic reactions in the subliminal evaluations of the self and others; but the involvement of consciousness with supraliminally presented stimuli may reduce this dissociation.

## Introduction

The self is a special psychological construct evolutionarily important to human beings, and has been extensively studied in its structure and content as well as motivational and affective implications [1]. One of the important topics is how processing self-stimuli affects one's conceptions of the self in terms of different qualities and attributes, i.e., one's self-evaluation.

It has been widely established that people evaluate self-related information in a way to maintain their self-esteem [2], [3], [4]. For example, indivdiuals typically judged the self more positively than they do to others [2], [5], [6], and the information that was more positive was thought as more self-descriptive [5], [7]. These effects have mostly been observed in implicit ways: Stimuli associated with the self, such as the letters in one's own name or the first person pronouns, were tended to be evaluated more positively than stimuli that were not [8], [9], [10]. Researchers also found faster responses in classifying one's self and positive attributes by key pressing in the Implicit Association Test (as a measurement of implicit self-esteem) [11], [12]. Recently, this so-called self-positivity effect has been found to extend to the self-face: Participants were more likely to judge attractive morphs (morphed self-face with an attractive other's face) rather than their actual faces or unattractive morphs as the self [13]. They also perceived morphs of their own faces as more trustworthy than morphs of others' faces [14].

Our study explored implicit self-evaluation elicited by face stimuli, but differed from past research in several important aspects.

First, we particularly focused on the subliminal self-evaluation, and adopted a novel method to elicit prolonged sublimimal processing of face stimuli. A common approach in the forementioned studies (such as studies on the name letter effect and the IAT) manipulates *supraliminally* presented self stimuli to be task-irrelevant, rendering the self-evaluative process unintentional or unrecognized. However, methodological disputes exist regarding whether perceivers are completely unaware of the mental content being tested, such as in the IAT [15], [16]. In other words, these procedures only ensure that the self-evaluation is not revealed by individuals' introspection, but does not necessarily mean that participants cannot introspectively access the evaluative processes, or at least, they might be aware that some characterisitcs of themselves were being assessed, which could contaminate the results to a certain extent. Studies considered to be more implicit employed the masked priming method, usually with less-than-50-ms presentation of self-related stimuli, including faces [17], names [18], or identity-relevant words [19]. The short exposure, such as the 17 ms in Spalding and Hardin's study [20], is assumed to create *subliminal* presentations of the stimuli, for participants are not able to report them consciously. Thus the influence of these short-exposed stimuli on participants' subsequent categorization tasks is considered to be out of awareness. However, recent research has begun to argue for the very need of attention resources for the priming effect as well as possible influences of participants' expectations or motivations in these traditional masked priming methods [21].

In the current study, we adopted a stricter approach to actualize *subliminal* presentation of the self-face using the Continuous Flash Suppression (CFS) paradigm to assess the self-evaluation below awareness. The CFS creates a reliable suppression of a low-contrast image presented to one eye of the participant, by flashing distinct Mondrian patterned noise images to the corresponding location of the other eye, and the interocular suppression elicited can last ten times or even longer than generated by other techniques such as binocular rivalry [22]. This would ensure the invisibility and unconsciousness for participants to the presented stimuli [22], [23], [24], and allow us to examine and validate the positivity nature of subliminal self-evaluation in a more stringent condition.

Secondly, unlike previous research on implicit self-evaluation that mostly relied on semantic materials [25], our study used face stimuli as primes. The rich information and special salience of the self-face for being a face and self-referential at the same time [26], [27], make it a good stimulus for studying self-related processing, especially self-evaluation. For example, ratings of one's own face image (compared with those of others) have been found to positively correlate with explicit embarrassment ratings and activation of brain regions related to self-evaluation [28]. Similarly, exposure to pictures of self and unattractive others (compared with pictures of self and attractive others) implicitly enhances activation in the brain areas involved in self-relatedness and reward processing [29].

We are also interested in subliminal other-evaluation, i.e., how participants evaluate others subliminally. We believe this would complement our understandings of behavioral manifests of the subliminal mental processes involved. Therefore we not only included the self-face but also faces of other people as subliminal primes to compare their effects on participants' subsequent valence judgments of positive and nagative personality trait words.

Past self-evaluation research, such as Spalding and Hardin's [20], was not designed to examine other-related evaluation, and therefore comparisons were made between self and generic non-self neutral stimuli. Even in studies that included “others” as primes, such as in Baldwin's [30], the focus was often on how the presence of these stimuli directly affected self-related evaluations and experiences (but see [31] for discussions of other-related evaluation). While Baldwin's study [30] suggests that internalizing opinions of specific others can affect self-evaluations, spontaneous, low resource-consuming social comparison is another major source of self-evaluation [32]. That is, people's self-image is often shaped according to the results of comparing with others when exposed to stimuli related to others [33], [34]. Thus comparing the self- and other-face in our study offered an opportunity to examine self-evaluation that may result from possible social comparisons.

In addition, our study examined indivdiual differences in subliminal self- and other-evaluation. We specifically focused on the role of one's self-esteem for two reasons. One is that self-esteem itself gives rise to different types of self-views: High self-esteem is characterized by a general fondness and love for oneself, whereas low self-esteem is associated with mildly positive or ambivalent feelings toward oneself [35], [36]. People with negative self-views (e.g., socially anxious, low in self-esteem, depressive) are less likely to show self-postivitiy than those with positive self-views [37], [38]. Self-esteem has also been found to modulate one's implicit self-evaluation under experimentally induced threats to one's self-concept. For example, responding to failure feedback or interpersonal rejection, participants high in self-esteem reported elevated liking for their name letters [39], automatically recruited thoughts about their personal strengths and suppressed thoughts about their weaknesses [40], whereas participants low in self-esteem automatically reacted with self-depreciation and withdrawal [41]. We wonder whether self-esteem modulates indivdiuals' subliminal self- and other-evaluation when there is no perceived threat to one's self.

To further explore the necessity of subliminal priming of self- and other-faces in trigerring specific patterns of self- and other-evaluations, we conducted Experiment 2 to examine implicit self-evaluation at the supraliminal level as a comparison.

To summarize, we primed participants with faces of their own and others' subliminally (Experiment 1) and supraliminally (Experiment 2) to investigate participants' implicit self- and other-evaluations indicated in a valence judgment task of personality traits. We also examined whether these evaluations would be modulated by participants' self-esteem at different levels of awareness.

## Experiment 1

### Methods

#### Participants

Forty-eight undergraduate students at Peking University participated in this experiment as paid volunteers. All were right-handed with normal or corrected-to-normal regular and stereo vision. Data from eight participants were excluded from analysis: Six participants failed to produce reliable suppression (see Procedure), and two had extremely lower performance accuracy in the valence judgment task (i.e., below 3 standard deviations of the mean accuracy of all participants). Results from the remaining 40 participants were included in the final analyses (13 men, 27 women; age range = 18–24 years, *M* = 22.5 years, *SD* = 2.1). Informed consent was obtained from each participant before this study, and this experiment was approved by the ethics review committee of the Department of Psychology, Peking University.

#### Materials

A facial picture of each participant, taken by a digital camera under the same lighting condition before the experiment, was used as the face stimulus for the “self” condition. Two sets of facial pictures were preselected, including eight males and eight females from students of similar age and social identity but unknown to the participants, but only the set of pictures of the same sex as the participant was presented in the “other” condition for that participant.

**Figure 1:**
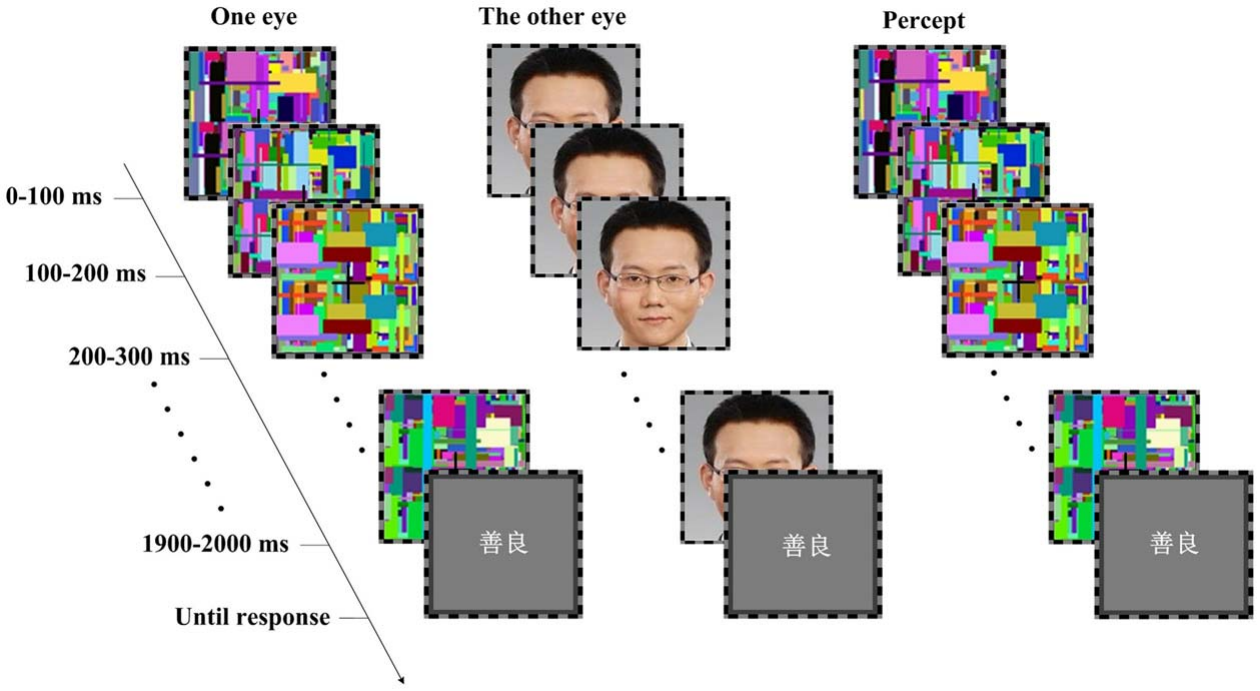
Schematic representation of the experimental paradigm for the main session in Experiment 1. In Experiment 2, the noise patches were replaced by the same face picture presented to the other eye.

All pictures showed neutral facial expression, and were processed using Adobe Photoshop to equalize the pupil-pupil distance with the vertical midline bisecting each face image. Each picture was then cropped so that only the head was shown in the image (see Figure 1), and resized to 130×130 pixels (visual angle 5.5°×5.5°). Then all modified images were matched on luminance (self: *M* = 3.82 cd/mm^2^, *SD* = 0.22; other: *M* = 3.84 cd/mm^2^, *SD* = 0.14; *t* (45) = −0.30, *p*>.1) and contrast (*M* = 0.36 for both the self- and other faces, based on the root-mean-square contrast), before framed against a gray background (110, 110, 110 RGB).

Noise images were the same size as face images, generated in sets in OpenGL 2.0 (Silicon Graphics International Corp., Fremont, California, US) prior to the experiment: Each set included 20 different Mondrian patterns, with each pattern containing randomly located squares of various sizes and colors. In each trial of the experiment, a set of the noise images were presented in succession at a canonical rate of 10 Hz to render the interocular suppression [22].

Two groups of two-character Chinese trait words, including 55 positive words (e.g., brave, kind-hearted) and 55 negative words (e.g., lazy, clumsy), with matched frequencies were selected from an established personality trait adjective pool [42].

#### Procedures

Participants took part individually in this experiment and went through a computer task with their heads supported by a chin rest at a viewing distance of 46 cm from the computer screen. They then completed a post-research questionnaire.

The computer task included a pre-test and a main session. The pre-test consisted of 100 Two-Alternative Forced-Choice trials (2AFC). Its administration was to safeguard the effectiveness of interocular suppression in the main session. Specifically, for half of the trials in this pre-test, in each trial a static face image, either self or other, was presented for 2 s to one eye of the participant with the dynamic noise simultaneously presented to the other eye. A mirror stereoscope was used to create the fused perception from images presented to both eyes, i.e., an interocular suppression of the face image. For the other 50 trials no face stimuli but only noise images were presented. Participants were informed before the session that each trial had a 50% probability to contain a face image, and they had to indicate “yes” or “no” on whether they thought a face had been presented by key pressing after each trial. If a participant's accuracy of such forced-choices significantly deviates from the chance level (*p*<.05, chi-square test), it means failure of suppression and the experiment would discontinue for that participant. Comparing the accuracy of participants' judgments in such a 2AFC task with the chance level has been widely used as a rigorous method to check for the presence of awareness in subliminal research [43], [44]. As a result, six participants with accuracy significantly higher than the chance level in their judgments were excluded from the study, ensuring that participants' data in the final analysis were indeed generated below awareness.

The main session of the computer task included 220 trials. Each trial (see Figure 1 for a graphic illustration) began with a 2-sec interocular suppression of face image (self or other) and dynamic noise images projecting to different eyes of a participant, same as in half of the trials in the pre-test session. Then a valenced personality trait word was presented binocularly to the participant, and s/he was asked to, as quickly as possible, press a key to indicate whether the word was positive or negative, and his/her reaction time was recorded.

**Figure 2:**
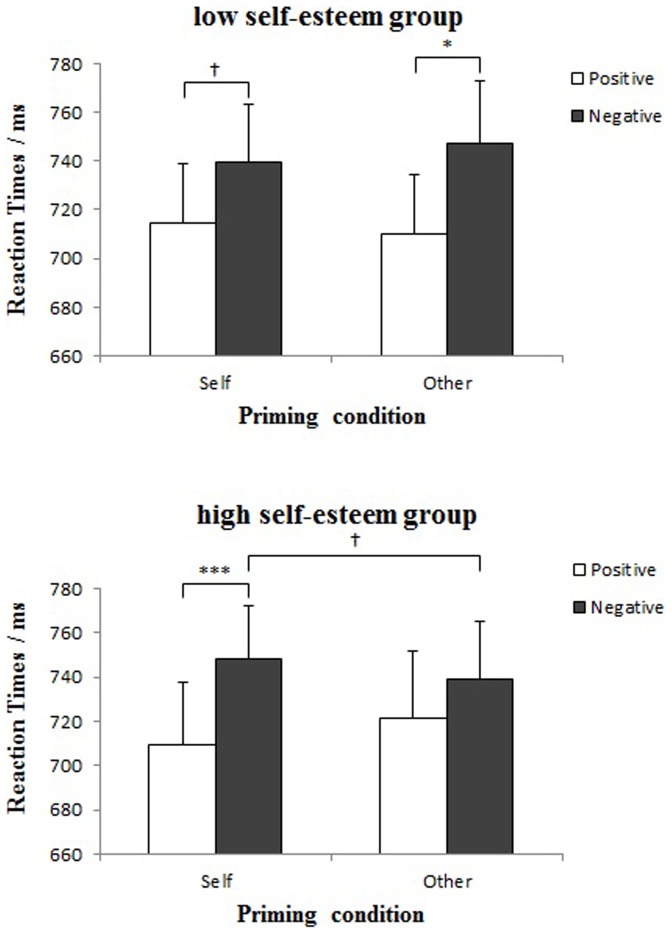
Mean reaction times to valenced words by face primes at different self-esteem levels. For this and the following figures, † *p*<.08, * *p*<.05, ** *p*<.01, *** *p*<.001, errors bars indicate standard error.

To further ensure the face stimuli were below participants' awareness in the main session, during presentations of the suppressed face images, participants were also instructed to press the spacebar whenever they saw anything else other than the dynamic noise images. For each participant, such a response occurred for less than 0.7% of total trials. These trials, and trials with participants' incorrect valence judgment of the trait word (<3.5% of the total trials), as well as trials with extremely long response times (>3 IQRs, <0.6% of the total trials) were excluded from data analysis.

After completing the computer task, participants finished the Chinese version of the Rosenberg Self-Esteem Scale (RSES) [45] embedded in filler items. This questionnaire has 10 items to be rated based on 4-point likert scales from 1 (Strongly disagree) to 4 (Strongly agree). The maximum possible score is 40. Higher scores indicates higher level of self-esteem. Based on the median split of their scores on this scale (Mdn = 30), participants were classified into high and low self-esteem groups.

### Results

Participants in the high self-esteem group reported significantly higher self-esteem scores on the RSES (*M* = 33.05, *SD* = 2.70) than the low self-esteem group (*M* = 27.45, *SD* = 3.15), *t* (38) = 6.03, *p*<.001.

Participants' reaction times were subjected to a mixed-measures ANOVA, with Valence (positive vs. negative) and Face (self vs. other) as within-participant factors, and Self-esteem (higher vs. lower) as a between-participant factor. Results revealed a significant main effect of Valence: It took participants longer time to react to negative than positive trait words, *F* (1, 38) = 16.65, *p*<.001, *η^2^* = .30 (see Figure 2).

More importantly, this effect was qualified by a significant Self-esteem×Face×Valence interaction, *F* (1, 38) = 5.53, *p* = .024, *η^2^* = .13 (see Figure 2). Simple effects analysis revealed that, participants low in self-esteem responded marginally faster to positive than negative trait words after primed with self-faces (*M*_positive_ = 714.2 ms, *M*_negative_ = 739.7 ms), *F* (1, 19) = 3.97, *p* = .061. But this pattern was significant after primed with other-faces (*M*_positive_ = 710.0 ms, *M*_negative_ = 746.9 ms), *F* (1, 19) = 7.40, *p* = .014. Meanwhile, these participants' reaction times to words of either valence did not vary between face primes (*p* = .555 for positive words, and *p* = .337 for negative words). On the other hand, participants high in self-esteem reacted significantly faster toward positive over negative words after self-face priming (*M*_positive_ = 709.6 ms, *M*_negative_ = 748.3 ms), *F* (1, 19) = 23.21, *p*<.001, but not after other-face priming (*M*_positive_ = 721.3 ms, *M*_negative_ = 739.3 ms), *F* (1, 19) = 2.76, *p* = .113. Although these participants' reaction times to positive words did not vary by face primes, *F* (1, 19) = 2.54, *p* = .127, their responses to negative words were marginally slower after primed with self- than other-faces, *F* (1, 19) = 3.50, *p* = .077. No other main effects or interactions were significant (all *p*>.1).

### Discussion

Results of Experiment 1 showed the modulating effect of self-esteem on subliminal self- and other-evaluation. Specifically, strong self-positivity reflected in the valence judgment task was only observed among participants high in self-esteem. They not only responded faster to positive than negative words following self-face primes, but also showed a tendency of inhibiting self-negativity (vs. other-negativity). Meanwhile, no positivity effect was found with other-face primes among these participants. However, participants low in self-esteem demonstrated a different pattern: a strong ‘other-positivity’ bias with faster responses toward positive than negative personality adjectives following subliminal other-face primes, and only a weak tendency of self-positivity. These findings indicate that the subliminal processing of one's own and others' faces gives rise to different response patterns in the implicit self- and other-evaluations between high and low self-esteem individuals.

Interestingly, while these results were in line with the supposed behavioral patterns of self-esteem, they seemed to be inconsistent with past research that suggests disassociations between explicit and implicit self-esteem [46], [47]. However, these past findings were often based on examining data from different types of measures, i.e., self-report esteem questionnaires vs. the IAT. We conducted Experiment 2 with a methodology that was comparable to Experiment 1 to further test the role of awareness in elicitng different patterns of self- and other-revaluations for people with different self-esteem levels.

## Experiment 2

Experiment 2 followed a more traditional approach to examine the role of self-esteem on participants' possible self-positivity bias. Specifically, we supraliminally presented participants the same materials as in Experiment 1, with the face images of their own and others as task-irrelevant stimuli followed by the same valence judgment task.

### Methods

#### Participants

Twenty-two undergraduate students at Peking University who did not attend Experiment 1 were recruited for Experiment 2 as paid volunteers (10 men, 12 women; age range = 18–24 years, *M* = 21.3 years, *SD* = 2.1). All were right-handed, with normal or corrected-to-normal regular and stereo vision. Informed consent was obtained from each participant before this study. As Experiment 1, this experiment was approved by the ethics review committee of the Department of Psychology, Peking University.

#### Procedures

The procedures of Experiment 2 were the same as that of Experiment 1 with two exceptions: 1) participants did not go through the pre-test, and 2) there were no noise images but participants viewed the supraliminal face-image binocularly (no rivalry) in each trial. Participants were divided into high and low self-esteem groups based on the median split of their scores on the RSES (Mdn = 30).

Since no participant's performance accuracy was significantly deviated from the overall mean, all participarnts' reaction times were included in the data analysis. As in Experiment 1, trials with extremely long reaction times (>3 IQRs) were excluded (<1%).

### Results

Same as Experiment 1, the high self-esteem group in Experiment 2 reported higher self-esteem scores (*M* = 33.45, *SD* = 2.25) than their low self-esteem counterparts (*M* = 26.64, *SD* = 3.80), *t* (20) = −6.82, *p*<.001. There was no significant difference in participants' self-esteem scores between Experiment 1 and 2, *t* (60) = .40, *p* = .72.

A mixed-measures ANOVA was conducted on participants' reaction times, with Valence (positive vs. negative) and Face (self vs. other) as within-participant factors, and Self-esteem (higher vs. lower) as a between-participant factor.

**Figure 3:**
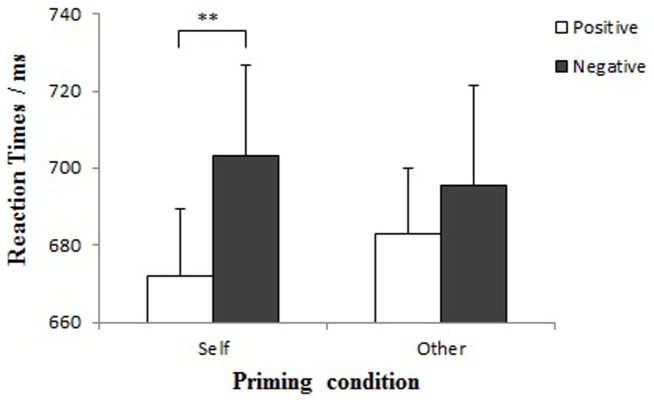
Mean reaction times to negative and positive words after conscious viewing of self- and other-faces.

Similar to Experiment 1, results showed a significant main effect of Valence: Participants responded faster toward positive words (*M* = 677.3 ms) than negative words (*M* = 699.3 ms), *F* (1, 20) = 6.89, *p* = .016, *η^2^* = .26, which was qualified by a significant Face×Valence interaction, *F* (1, 20) = 4.70, *p* = .042, *η^2^* = .19 (Figure 3). Specifically, participants responded to positive words significantly faster than to negative words after viewing self-faces (*M*_positive_ = 671.8 ms, *M*_negative_ = 703.1 ms), *F* (1, 21) = 9.87, *p* = .005, but not so after viewing other-faces (*M*_positive_ = 682.7 ms, *M*_negative_ = 695.6 ms), *F* (1, 21) = 2.27, *p* = .147. Meanwhile, shorter reaction times following self- than other-face primes were found for positive words, *F* (1, 21) = 7.60, *p* = .012. No other effects, including the ones involving the self-esteem factor, were significant, all *p*>.5.

### Discussion

When the face stimuli were presented supraliminally in Experiment 2, we did not find the modulating effect of self-esteem on implicit self- and other-evaluation. Rather, all participants demonstrated the self-positivity bias, with faster responses to positive than negative words after self-face primes. These results are consistent with previous studies on the dissociation of implicit self-evaluation and explicit self-esteem [46], [47]. Participants did not show any positivity effect after viewing other faces.

## General Discussion

We conducted two experiments to examine how participants evaluated themselves as well as similar unknown others as reflected by their responses to valenced personality traits after subliminally or supraliminally primed with their own and unknown others' faces. We also investigated the modulating effect of participants' self-esteem on their valence judgments.

With a new method to ensure the subliminal self-processing, we found varied patterns of self- and other-evaluations that were modulated differently by one's self-esteem at different levels of awareness. Results from Experiment 1 on subliminal evaluations demonstrated prominent other-positivity (and only weak self-positivity) among participants low in self-esteem, but significant self-positivity among participants high in self-esteem. However, self-esteem did not modulate participants' implicit self-evaluation in Experiment 2, which is consistent with past findings of the dissociated patterns between implicit self-evaluation and explicit self-esteem with supraliminal presentation of self-related stimuli [10], [48] or masked priming methods [18], [20].

We believe that the different characteristics of self-processing elicited by supraliminal and subliminal face stimuli accounted for the above results. Under the interocular suppression, there was no involvement of conscious effort in the responses of participants, and thus these individuals of high and low self-esteem levels might have demonstrated their different automatic, “default” response patterns. Similarly, it has been found that when confronting self-threats inividuals with high self-esteem showed amplified self-positivity [39] and those with low self-esteem showed self-depreciation [41]. It may be because these responses all had an automatic nature and rarely involved controlled processes.

On the other hand, processing supraliminal self-related stimuli is a dual-processing that recruits both automatic and controlled processes [49]. In other words, unlike minimized in Experiment 1 due to the prolonged subliminal processing, the controlled regulation might manifest in Experiment 2, even when the relevance of the face stimuli was only implicitly established (but supraliminally presented). As a result, even participants with lower self-esteem in our study demonstrated similar self-positivity but indifferent responses toward other-evaluation, as participants with higher self-esteem did. We further speculate that part of the reason for the lack of consistence in past research on implicit self-evaluation and explicit self-esteem might be due to the research methodology: with different methodologies, participants may vary in the degree they would be able to adopt the controlled processes.

An interesting finding in our study was that at the subliminal level individuals with lower and higher self-esteem mainly differentiated from each other on their evaluations toward others, and less on demonstrating self-positivity. Therefore examining participants' other-evaluation contributes to understanding the complexity of one's self-evaluation. Using a masked priming paradigm, Wentura et al. [18] also revealed the modulating effect of self-esteem on one's implicit self-evaluation with presentations of both self- and other-stimuli (i.e., name initials). However, responses to negative words primed by others were incorporated in their estimation of one's positive self-regard, and they did not analyze self- and other-evaluations separately. In addition, although enhanced self-view could be achieved by lowing others [50], our study across both experiments did not reveal negative reactions toward others, suggesting that additional social prompts may be needed to activate that mechanism.

Future studies should continue to explore implicit self- (and other) evaluation at different levels of awareness, for example, to identify neural evidence for the dissociative subliminal and supraliminal self-evaluation at different levels of self-esteem. In addition, since specific stimuli (events or images) could give rise to automatic negative thoughts, affects, and physiological symptoms for low self-esteem individuals [51], it is worth further examining how different types of stimuli may impact implicit self-evaluation modulated by self-esteem.

## Conclusions

With an interocular suppression technique, our study extends previous research on self-processing in three aspects: 1) we show the differences of implicit self-evaluations at the subliminal and supraliminal levels; 2) we confirm that the role of self-esteem in self-evaluation depends on the unawareness of the ‘self’ involved; 3) we offer a more complete picture in understanding how individuals view themselves in the context of possible social-comparisons by including other-evaluation in our investigation.
